# Gerstmann's syndrome and unilateral optic ataxia in the emergency department

**DOI:** 10.1590/1980-57642016dn11-040018

**Published:** 2017

**Authors:** Breno José Alencar Pires Barbosa, Marcelo Houat de Brito, Júlia Chartouni Rodrigues, Gabriel Taricani Kubota, Jacy Bezerra Parmera

**Affiliations:** 1 Department of Neurology, Hospital das Clínicas, University of São Paulo Medical School – São Paulo, SP, Brazil.

**Keywords:** Gerstmann syndrome, stroke, carotid artery diseases, agnosia, síndrome de Gerstmann, acidente vascular cerebral, doenças da artéria carótida, agnosia

## Abstract

A 75-year-old right-handed woman presented to the emergency department with simultanagnosia and right unilateral optic ataxia. Moreover, the patient had agraphia, acalculia, digital agnosia and right-left disorientation, consistent with complete Gerstmann's syndrome. This case highlights the concurrence of Gerstmann's syndrome and unilateral optic ataxia in the acute phase of a left middle cerebral artery stroke.

## RESUMO.

Uma paciente de 75 anos e destra se apresentou à sala de emergência com quadro de simultanagnosia e ataxia óptica unilateral à direita. Além disso, a paciente tinha agrafia, acalculia, agnosia digital e desorientação direita-esquerda, compatíveis com a síndrome de Gerstmann. O presente caso destaca a ocorrência simultânea da síndrome de Gerstmann com ataxia óptica unilateral na fase aguda do acidente vascular cerebral da artéria cerebral média esquerda.

## CASE REPORT

A 75-year-old right-handed woman presented to the emergency department with headache and mental confusion 6 hours before admission. She had 5 years of formal education and was able to read and write perfectly. There was no prior medical history other than tobacco use for 50 years. Her family reported that she initially complained of difficulties in handling objects with her right hand and they noticed she was confused.

Initial evaluation revealed a blood pressure of 220 × 120 mmHg and a mild pronator drift of the right upper limb. She was alert and oriented, with no global attention or language abnormalities. Testing was positive for mild right spatial neglect with sensory extinction alone. Her score on the National Institute of Health Stroke Scale (NIHSS) was 1. The patient underwent initial stabilization and a Computed Tomography (CT) scan, which revealed only a residual calcified lesion in the left post-central gyrus. Interestingly, a more detailed cognitive evaluation revealed simultanagnosia (she could not integrate parts of the Boston Cookie Theft Picture or small letters into a global form) and unilateral right optic ataxia (video). Moreover, the patient had agraphia, acalculia, digital agnosia and right-left disorientation, consistent with complete Gerstmann's syndrome (video).

**Video:** Testing optic ataxia (first part) and Gerstmann's syndrome (second part). The patient was also tested for acalculia separately with written numbers (not shown) (available in www.demneuropsy.com.br).

**Figure 1:**
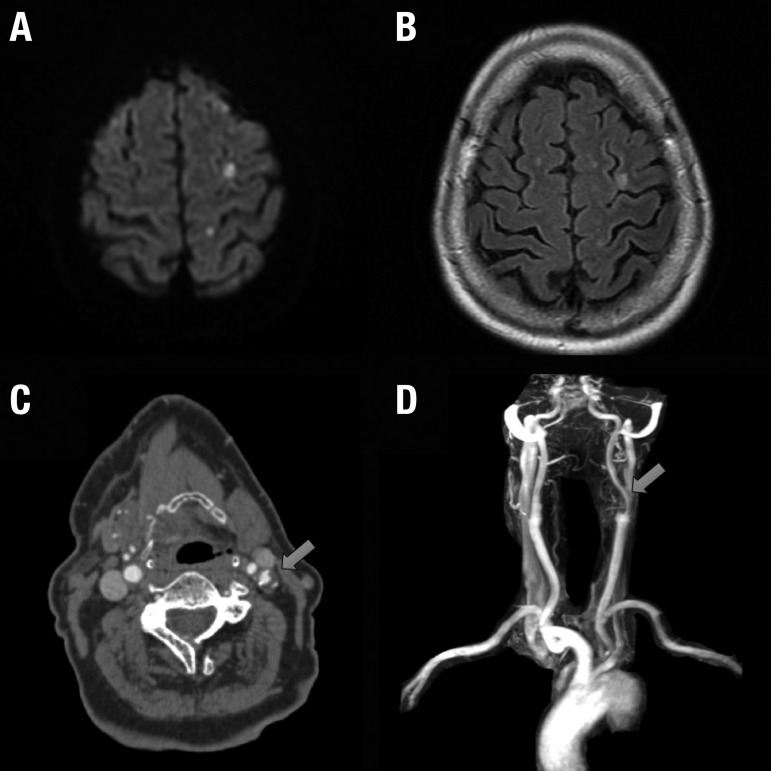
Diffusion-weighted imaging (A) and T2-FLAIR (B) MRI showing multiple small hyperintense foci in the middle frontal and post central gyri. Computer Tomography Angiography (C) and MR Angiography (D) showing mild atheromatosis in both carotid arteries. The left atherosclerotic plaque is soft and ulcerated, causing vessel stenosis of approximately 50%.

CT angiography showed diffuse atheromatosis, with moderate stenosis of 50% in the left carotid bulb due to an ulcerated plaque (Figure 1). Diffusion-Weighted Imaging MRI revealed small hyperintense lesions in the territory of the left middle cerebral artery, predominantly posterior and cortical (Figure 1). Serial electroencephalogram testing was negative for epileptiform activity, but there was a focal electrical slowing in the left hemisphere. After a complete neurovascular work-up, we concluded the patient had a stroke due to an evident large artery atherosclerosis mechanism.

The patient was treated with dual antiplatelet therapy and a high-potency statin at standard doses for secondary prevention. All cognitive deficits resolved spontaneously within 3 days of admission and she was discharged at day 4 with a modified Rankin-scale of 1 for outpatient follow-up, rehabilitation and smoking cessation.

## DISCUSSION

This case highlights the concurrence of Gerstmann's syndrome, right spatial neglect and unilateral right optic ataxia in the acute phase of a left middle cerebral artery stroke. These syndromes have been described in association with a wide range of neurological conditions, including neurovascular diseases.^1^^-^ 6

Gerstmann's syndrome is defined in the presence of finger agnosia, right-left disorientation, agraphia and acalculia. Agnosia is the inability to recognize external information in the absence of substantial sensory deficits. Agraphia and acalculia reveal impairment in the ability to write and manipulate numbers, respectively. Zukic et al.^3^ observed 194 acute stroke cases and reported the finding of alexia, agraphia and acalculia alone, or in combination, in up to 30.4% of cases. However, complete Gerstmann's syndrome was only reported in less than 5% of the patients in this series.

Despite some controversies with regard to Gerstmann's syndrome topographic value,^7^ there is reasonable agreement that the most probable location involves the left angular gyrus with some subcortical extension. João et al.^6^ recently reported an interesting case of transient Gerstmann's syndrome following a left middle cerebral artery infarct with lesion to the inferior frontal lobe and insula. This case illustrates the occurrence of Gerstmann's syndrome even in cases where the angular gyrus is spared. This is probably explained by a disconnection of association fibers connecting the frontal and parietal lobes (i.e. superior longitudinal and arcuate fasciculus), as illustrated by tractography studies.^8^

Optic ataxia is the inability to accurately reach a target under visual guidance. The lesions for optic ataxia are most probably related to the parieto-occipital border zones and represent a disorder of the sensory-guided motor behavior of the posterior cerebral cortex, with contributions from the supplementary motor area in the frontal lobes.^9^

Regarding the neural systems subserving visual processing, the outputs from primary and secondary visual cortex are traditionally divided into the dorsal and ventral streams. Whereas the ventral stream is associated with transforming visual input into representations of objects' characteristics, the dorsal stream mediates navigation and the visual control of actions directed at objects.^10^ There is, however, new evidence that the dorsal stream is further subdivided into a ventro-dorsal and dorso-dorsal system. The subject was recently revisited by Binkofsky and Buxbaum.^11^ On one hand, the ventro-dorsal stream plays a major role in processing object use representation, such as the use of tools (*use system*). On the other hand, the dorso-dorsal stream is the visual pathway for action, such as reaching targets with various locations in space (*grasp system*). A lesion to this system would result in optic ataxia.

An interesting finding in our case is the concurrence of unilateral right optic ataxia and simultanagnosia. Nagaratnam et al.^12^ reported 5 cases of optic ataxia due to unilateral stroke. The lesion was located in the parieto-occipital regions in three patients but were anatomically further forwards in the remaining two cases. The optic ataxia was bilateral in one patient, but the deficit was more prominent on the contralateral side of the cerebral lesion.

The lateralization of optic ataxia (*grasp system*) with a relatively preserved ability to use objects (*use system)* in this case illustrates the complexity of the dorsal system in the left cerebral hemisphere in a clinical background. The absence of a functional imaging study in this case is a limiting factor, since it could have demonstrated regional reduced activity and further corroborated our discussion.

In conclusion, since these symptoms may be transient, our case highlights the importance of testing for cognitive syndromes such as Gerstmann's and Balint's in suspected cases in the acute phase of neurovascular conditions.
